# Characterizing gaze position signals and synthesizing noise during fixations in eye-tracking data

**DOI:** 10.3758/s13428-020-01400-9

**Published:** 2020-05-29

**Authors:** Diederick C. Niehorster, Raimondas Zemblys, Tanya Beelders, Kenneth Holmqvist

**Affiliations:** 1 Lund University Humanities Laboratory and Department of Psychology, Lund University, Lund, Sweden; 2 Šiauliai University, Šiauliai, Lithuania; 3 Department of Computer Science and Informatics, University of the Free State, Bloemfontein, South Africa; 4 Institute of Psychology, Nicolaus Copernicus University in Torun, Torun, Poland; 5 Department of Psychology, Regensburg University, Regensburg, Germany

**Keywords:** Eye tracking, Precision, Data quality, Fixational eye movements, Power spectrum, Signal color

## Abstract

The magnitude of variation in the gaze position signals recorded by an eye tracker, also known as its precision, is an important aspect of an eye tracker’s data quality. However, data quality of eye-tracking signals is still poorly understood. In this paper, we therefore investigate the following: (1) How do the various available measures characterizing eye-tracking data during fixation relate to each other? (2) How are they influenced by signal type? (3) What type of noise should be used to augment eye-tracking data when evaluating eye-movement analysis methods? To support our analysis, this paper presents new measures to characterize signal type and signal magnitude based on RMS-S2S and STD, two established measures of precision. Simulations are performed to investigate how each of these measures depends on the number of gaze position samples over which they are calculated, and to reveal how RMS-S2S and STD relate to each other and to measures characterizing the temporal spectrum composition of the recorded gaze position signal. Further empirical investigations were performed using gaze position data recorded with five eye trackers from human and artificial eyes. We found that although the examined eye trackers produce gaze position signals with different characteristics, the relations between precision measures derived from simulations are borne out by the data. We furthermore conclude that data with a range of signal type values should be used to assess the robustness of eye-movement analysis methods. We present a method for generating artificial eye-tracker noise of any signal type and magnitude.

## Introduction

Eye-tracking recordings are used in many fields of science, often to study where participants look or how their eyes move. For screen-based experiments using only static stimuli, eye-tracker data are often classified into two types of episodes, fixations (periods during which the participant continuously looks at a specific location on the screen) and saccades (periods during which gaze rapidly shifts to another position on the screen). See Hessels et al., (2018) for an in-depth discussion of fixation and saccade definitions. In this paper, we examine data quality during fixations to positions on a screen.

**Fig. 1:**
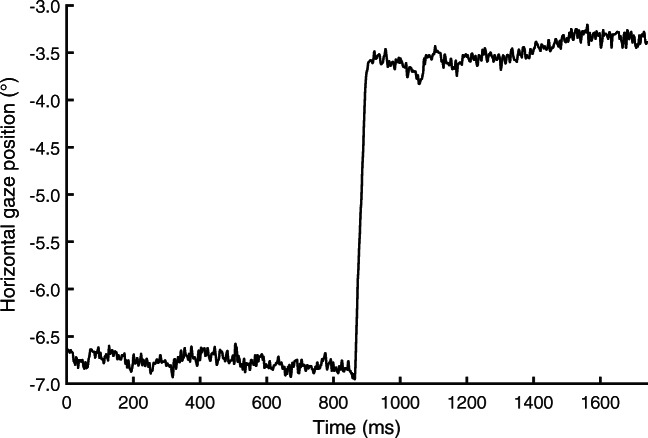
Gaze position data example. Example data segment showing two fixations interleaved by a saccade at about 900 ms, recorded with an SR EyeLink 1000Plus

Figure 1 plots example eye-tracking data showing two fixations interleaved by a saccade. As can be seen, even during a fixation when the participant’s gaze remains fixed on a certain location, the recorded gaze position is not constant but appears to vary. In eye-movement data, these variations are thought to arise from at least two sources, 1) noise inherent in the measurement device, and 2) the rotations of the eyeball itself, such as tremor, drift, and microsaccades (Ratliff and Riggs, 1950; Collewijn & Kowler, 2008; Martinez-Conde et al., 2004; Rolfs, 2009). In this paper, we will refer to these two signal components as *measurement noise* and *fixational eye movements*, respectively. Researchers interested in small fixational eye movements such as microsaccades and drift should have a keen interest in ensuring that the magnitude of measurement noise in their eye trackers’ output is low enough that it does not obscure these small eye movements (Ko et al., 2016). As measurement noise gets larger, it will make small saccades undetectable, thereby altering the distributions of saccade, fixation, and other event measures calculated from the data (Holmqvist et al., 2012).

To quantify the magnitude of variability in eye-tracking data during fixations (we will refer to this as *signal magnitude* throughout the paper), researchers have developed several measures that are often referred to as “precision measures”. The most well-known of the precision measures are the root mean square of the displacement between successive gaze position samples (RMS-S2S) and the standard deviation of the gaze position samples (STD). These two measures however do not provide consistent assessments of signal magnitude. This becomes clear when examining a ranking of eye trackers by their precision (Table 1, computed from the data recorded for this study). In this table, it is seen that ranking eye trackers by precision as assessed by RMS-S2S yields a different ordering than when ranking by STD. It should also be noted that while some systems show an RMS-S2S value that is much smaller than the STD value (e.g., the EyeLink 1000Plus and the SMI RED250), other systems show an RMS-S2S value that is larger than the STD value (the Tobii TX300 and the Tobii X2-60). The finding that RMS-S2S and STD lead to different orderings when ranking eye trackers by precision indicates that each measure provides only a limited view of the precision of an eye tracker, and that neither is the gold standard measure of signal magnitude.

**Table 1:** Eye tracker ranking by precision. Ranking of five eye trackers by increasing median precision, given as both RMS-S2S and STD. Note how the SMI RED250 and the Tobii TX300 switch places in the ranking when precision is assessed using STD instead of RMS-S2S

RMS-S2S (^∘^)		STD (^∘^)	
SR Eyelink 1000Plus	0.0187	SR Eyelink 1000Plus	0.0690
SMI RED250	0.0734	Tobii TX300	0.2311
SMI RED-m	0.2906	SMI RED-m	0.3362
Tobii TX300	0.2994	SMI RED250	0.3429
Tobii X2-60	0.5483	Tobii X2-60	0.4269

**Fig. 2:**
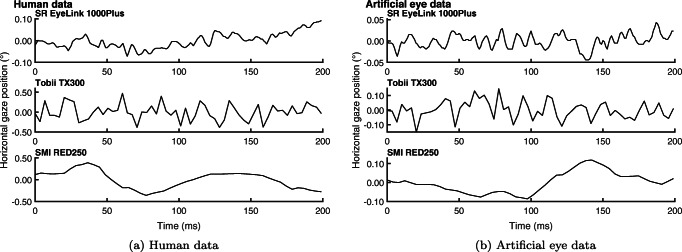
Gaze position data examples. For three trackers, example 200 ms segments from a fixation recorded from humans (left panel) and example 200 ms segments of data recorded with an artificial eye (right panel). The 1000 Hz EyeLink and 250 Hz SMI data in the top and bottom rows have lower RMS-S2S than STD values and look smooth. For the 300 Hz Tobii data in the middle row, the RMS-S2S values are larger than the STD values and the data looks less smooth and more randomly distributed than for the other two eye trackers

In this paper, we posit that the RMS-S2S and STD measures do not simply reflect the magnitude of variability in the eye-movement signal, despite being used on the specification sheets of eye trackers to indicate their noise level.^1^ Instead, we propose that RMS-S2S and STD values are also dependent on the *signal type* in eye-tracking data, and that signal type affects these two measures differently. Different signal types are readily seen when examining the horizontal gaze position time series recorded from humans (Fig. 2a) or from artificial eyes (Fig. 2b). While the data from the Tobii TX 300 (middle panels) contain large sample-to-sample steps and appear randomly distributed around a central point, the data from the SR Eyelink 1000 Plus and SMI RED250 appear much smoother and more similar to a random walk. Sample-to-sample displacement is lower in the smoother signals than in the more random-appearing signals of the Tobii TX300, which corresponds to lower RMS-S2S values for the smoother signals than for randomly distributed signals of the same spatial extent.

Understanding the nature of the signal provided by eye trackers is not only of esoteric theoretical interest; it also has further use beyond comparing eye-tracker data quality. Besides being of great relevance for studies of fixational eye movements (e.g., Bolger et al., 1999; Collewijn & Kowler 2008; Ko et al., 2016; see Niehorster et al., 2020b, for a discussion) and of the dynamics of gaze behavior (Aks et al., 2002; Coey et al., 2012; Wallot et al., 2015), being able to characterize the types of signals provided by contemporary video-oculography (VOG) eye trackers also has a bearing on how event classification algorithms are developed and evaluated. To assess whether the events (such as fixations and saccades) labeled by a classification algorithm remain stable under different noise magnitudes, it is starting to become common practice (e.g., Hessels et al., 2016; Zemblys et al., 2017) to add artificial noise at different magnitudes to the gaze position signals used for algorithm evaluation. To ensure that such evaluations reveal expected algorithm performance when classifying actual noisy gaze position signals, it is important to know what types of signals are found in eye-tracking data, and thus what types of noise should be added during these evaluations. There has been a debate about whether the appropriate method is to take recorded measurement variability and amplify it as Hessels et al., (2016) did, or instead, as Zemblys et al., (2017) did, by adding white noise of various magnitudes on the basis of the hypothesis that the measurement noise in all eye trackers is white (as reported by Wang et al., 2016, but see Blignaut and Beelders 2012 and Niehorster et al., 2020b, for diverging findings).

### Aims of this paper

Three over-arching issues arise from the inconsistencies in the literature reviewed above. First, the review above highlights that the concept of precision, as well as the measures thereof, are not yet fully understood. Second, it appears that the RMS-S2S and STD measures of precision may be differently affected by the signal type. Finally, it is unclear what type of noise should be added when developing and testing event classification algorithms.

To resolve these issues and develop an understanding of how signal type interacts with the RMS-S2S and STD precision measures, in this paper we analyze how these precision measures are related to each other and to the temporal spectrum content of the signal. To do so, we develop two new complementary measures characterizing the gaze position signal provided by the eye tracker that unambiguously indicate the magnitude of signal variability independent of the signal’s type, and orthogonally the type of the signal independent of its magnitude. We hypothesize that the new signal type measure is systematically related to the temporal spectrum content of the signal, and that both these measures can thus be used to assess the spectral color of the eye-tracker data.

In the Results section, we then first investigate the behavior of the various precision measures and the relationship between these measures using simulated signals that are generated with a new noise synthesis method that is also presented in the paper. We then furthermore apply the various precision measures to analyze recordings of human and artificial eyes made with five VOG eye trackers. Specifically, we perform correlational analyses revealing the first order relations between the measures examined in this study, and also perform an in-depth investigation of the relation between the new signal type measure and the temporal spectrum content of the signal.

Some of this material has previously been presented in Holmqvist and Andersson (2017, pp. 179–182). Furthermore, the data used in the empirical analysis sections of this paper are also used for parallel analyses in Niehorster et al., (2020b), which develops an understanding of the signal characteristics of various eye trackers. Specifically, the investigation in Niehorster et al., (2020b) provides insight into whether the apparent fixational drift that is often observed in the gaze position signals recorded with contemporary video-oculography eye trackers indeed reflects fixational eye movements or is instead largely due to the effect of filters in the eye tracker.

## Existing precision measures

In this paper, *precision measure* is used to refer to a measure that quantifies a certain aspect of the fluctuations in a gaze position signal observed during a fixation. There are at least three aspects of signals that are investigated in the literature that will be discussed in this paper. A first category of measures used by eye-movement researchers is intended to quantify the overall *signal magnitude* in gaze position data, such as by means of the RMS-S2S and STD measures. A second aspect of a signal is the statistical *distribution* that its values follow (e.g., Gaussian). Third, there exist measures of the *type* of the signal, such as its spectral color (e.g., white or pink).

In this section, we review precision measures from each of these three categories. The measures of signal magnitude and signal type are used for most of the analyses in this article, while signal distribution is discussed because of its role in the synthesis of artificial noise. Indeed, in the “Noise synthesis” section below we show that these aspects of a signal form independent, orthogonal categories.

### Signal magnitude

Following the International Vocabulary of Metrology (BIPM et al., 2012), an important aspect of data quality is the precision of a signal. In the case of eye-tracking data, precision is defined as the closeness of a set of repeated gaze position measurements obtained under identical conditions (i.e., obtained from an eye that has not rotated) (BIPM et al., (2012), p. 22, see also Niehorster et al., (2020a)). Precision complements accuracy, another important measure of data quality that refers to the closeness of the gaze position indicated by the eye tracker to the actual gaze position of the participant. This section discusses measures that operationalize the concept of signal magnitude. There are many measures in the literature that are used to characterize signal magnitude. Here we will discuss the three commonly used measures, RMS-S2S, STD and BCEA. For an overview of other measures of the precision of gaze position signals, the reader is referred to Holmqvist and Andersson (2017, Chapter 6 and pp. 503–515).

#### RMS-S2S

A common measure of signal magnitude is the root mean square of the displacement between successive gaze positions. This measure is often referred to as simply RMS, but is more correctly called RMS-S2S. For a sequence of *n* gaze position samples, the RMS-S2S is defined as

1 $$ \begin{array}{@{}rcl@{}} \text{RMS-S2S} &=& \sqrt {\frac{1}{n-1} \sum\limits_{i=1}^{n-1} {\theta_{i}}^{2}}\\ &=& \sqrt{\frac{1}{n-1} \sum\limits_{i=1}^{n-1} \left( (x_{i+1} - x_{i})^{2} + (y_{i+1} - y_{i})^{2}\right)}\\ \end{array} $$

where *x* and *y* refer to the horizontal and vertical components of recorded gaze position, respectively, and *𝜃* is the distance between successive gaze positions. Assuming a constant sampling frequency, the RMS-S2S of a segment of gaze position data is closely proportional to its average *velocity* if sample-to-sample variation is low compared to the average velocity of the segment. As such, RMS-S2S may serve as an indication of the velocity of the signal during fixations. This makes it a good candidate measure for assessing what the slowest eye movement is (e.g., a small saccade) that would be differentiable from the background noise in a gaze position signal (see, e.g., Holmqvist and Blignaut 2020). Possibly for this reason, RMS-S2S has become the de-facto measure of the precision of eye-tracking systems. Manufacturers report RMS-S2S in their eye-tracker specifications; it is used in comparisons of eye trackers (e.g., Nyström et al., 2013), and research has examined how fixation and saccade classification are affected by increasing signal magnitudes as reflected by increasing RMS-S2S values (e.g., Holmqvist et al., 2012; Hessels et al., 2016; Zemblys et al.,^2017^).

There are however problems with RMS as a measure of precision. The upper row and especially the lower row of Fig. 2 show a few example fixations for which the signal magnitude as measured by RMS-S2S is small, but the gaze position signals spread over a large area. Finding this kind of data led Blignaut and Beelders (2012) to suggest that RMS-S2S is unsuitable as a precision measure because large spatial dispersion and extensive systematic trends can be present in the data even though RMS-S2S indicates a low signal magnitude. In other words, RMS-S2S captures only one aspect of variability in eye-movement recordings, the magnitude of displacement between subsequent gaze samples, but it does not reflect aspects of the data over larger time scales, such as its spatial spread.

Insensitivity to spatial spread is not the only potential problem with RMS-S2S. RMS-S2S is also largely insensitive to infrequent large and brief excursions of the gaze position signal (spikes) in otherwise low-noise data (Blignaut & Beelders, 2012). While RMS-S2S would indicate a low signal magnitude for such data, the occasional spike could cause many fixation and saccade classification algorithms to return unwanted results. RMS-S2S has also been reported by Blignaut and Beelders (2012) to vary for the same data at different sampling frequencies, which led these authors to conclude that RMS-S2S cannot be used to compare the signal magnitudes of eye trackers that record at different sampling frequencies. Part of this problem could possibly be alleviated by computing the RMS of sample-to-sample steps after first scaling the steps by the sampling frequency of the signal to recover signal velocity. Further evaluation of this scheme is however required.

#### Standard deviation

Another common measure of signal magnitude is the standard deviation (STD). For a sequence of *n* gaze position samples, STD is defined as

2 $$ \begin{array}{@{}rcl@{}} \text{STD} &=& \sqrt{\frac{1}{n} \sum\limits_{i=1}^{n} \left( (x_{i} - \overline{x})^{2} + (y_{i} - \overline{y})^{2}\right)}\\ &=& \sqrt{\text{STD}_{x}^{2}+\text{STD}_{y}^{2}} \end{array} $$

where *x* and *y* refer to the horizontal and vertical components of the recorded gaze position, respectively, and $\overline {x}$ denotes the mean of sequence *x*. STD defined thusly is a measure of dispersion around the centroid of a sequence of gaze positions, i.e., a 1D measure characterizing the radial *extent* of the signal. Thus, in contrast to RMS-S2S, STD indicates the spatial spread of a sequence of gaze positions. Furthermore, STD is relatively insensitive, compared to RMS-S2S, to displacement between successive gaze positions (Blignaut & Beelders, 2012) and thus does not capture the velocity aspect of variability in eye-tracking data.

Note also that since $\text {RMS}_{x}^{2}=\text {STD}_{x}^{2}+\overline {x}^{2}$, RMS-S2S is equal to the STD of distances between successive gaze positions if the sequence of distances has a mean of zero.

#### Bivariate contour ellipse area

The bivariate contour ellipse area (Crossland & Rubin, 2002) is a measure of the area covered by a sequence of gaze position samples. It is defined as

3 $$ \text{BCEA} = 2k\pi\sigma_{x}\sigma_{y}\sqrt{1-\rho^{2}}  $$

where *σ*_*x*_ and *σ*_*y*_ denote the standard deviations of the gaze position sequence in the *x* and *y* directions, respectively, and *ρ* is the Pearson correlation coefficient between the sequences of recorded *x* and *y* gaze position samples. The constant *k* depends on the parameter *P* and determines the size of the BCEA ellipse in terms of the fraction *P* of measured positions that are within its contours. *k* is given by $k=- \log (1-P)$. *k* was set to 1 for this study (*P* = 1 − *e*^− 1^ = .632). Following Blignaut and Beelders (2012), we used the square root of the BCEA value in this study ($\sqrt {\text {BCEA}}$) to obtain a one-dimensional quantity that is directly comparable to STD, the other measure of spatial extent considered here.

It may be of interest to recover the ellipse underlying the BCEA measure, for instance to test whether spread in the signal is isotropic and if not, how it is oriented. These measures are furthermore of interest in this paper because the noise synthesis method presented below allows for the generation of such anisotropic signal segments. As per Niehorster et al., (2017), recovery of the BCEA ellipse can be achieved through factorization of the 2x2 covariance matrix of the measured *x*- and *y*-coordinates,

4 $$ \boldsymbol{V}_{\boldsymbol{xy}} = \left[\begin{array}{cc} {\sigma_{x}^{2}} & \rho\sigma_{x}\sigma_{y}\\ \rho\sigma_{x}\sigma_{y} & {\sigma_{y}^{2}} \end{array}\right] $$

through eigenvalue decomposition using

5 $$ \boldsymbol{V}_{\boldsymbol{xy}} = \boldsymbol{Q{\Lambda} Q^{-1}}  $$

This yields the diagonal matrix **Λ** = diag(*λ*_1_,*λ*_2_), where *λ*_1_ and *λ*_2_ are the eigenvalues of ***V***_***xy***_ which correspond to the squared lengths of the principle axes of the ellipse underlying the BCEA measure when *k* = 1 in Eq. 3. Anisotropy of the signal can then be assessed by computing the aspect ratio of the ellipse,

6 $$ AR = \sqrt{\frac{\lambda_{1}}{\lambda_{2}}} $$

Equation 5 also yields the 2 x 2 matrix $\boldsymbol {Q}=\left (\begin {array}{cc} q_{11}&q_{12}\\q_{21}&q_{22} \end {array}\right )$ of eigenvectors of ***V***_***xy***_ from which the orientation *𝜃* of the major axis of the ellipse is recovered by

7 $$ \theta = \tan^{-1}\frac{q_{21}}{q_{11}} $$

Note that the above procedure is equivalent to preforming a principal component analysis (PCA) of the gaze position segment. As such, it may be of interest to generalize the analysis through the use of independent component analysis (ICA), which allows for investigations focusing on higher-order moments in the gaze position segment. These techniques are not further explored in this paper.

### Signal distribution

Signals can take on various probability density distributions depending on the generating process, such as Gaussian, Rayleigh, or Poisson (Scherzer et al., 2009). We think it is a reasonable hypothesis that the signal recorded by an eye tracker (in absence of physical eye rotations) follows a Gaussian distribution. The logic is that we deem it likely that the measurement noise in VOG eye-tracking data results to a significant extent from the cumulative effect of independent noise in each of the many pixels of the eye image, which by the central limit theorem could be expected to generate Gaussian variation in the derived output signal. An understanding of the statistical distribution that best describes noise in eye-movement data is an important aspect for being able to generate realistic noise for the assessment of event classification algorithms.

**Fig. 3:**
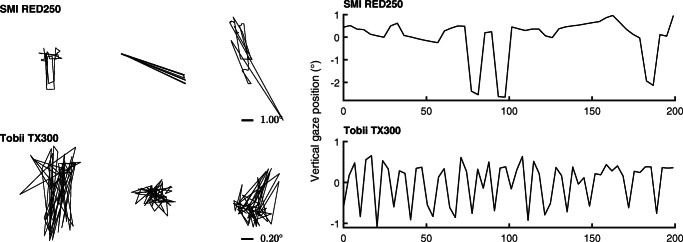
High-frequency oscillation examples. *Left panel*: example 200 ms segments from three fixations recorded from humans showing high-frequency oscillation (HFO) signals. The *right panel* shows the vertical component of the leftmost data segment in the left panel over time. HFO signals are characterized by having distances between successive gaze positions that can be almost as large as the range of the whole data segment. In a video-based eye tracker, such data may for instance result when one or more other bright spots in the eye image compete with the corneal reflection of the infrared illuminator (Holmqvist & Andersson, 2017, p. 138). Note that the scale at which data is visualized differs for each eye tracker. Data in the left panel were scaled per row for illustration purposes. The scale of the signals is therefore indicated for each eye-tracker’s data

However, eye-tracking data is not always well described by a Gaussian distribution. Specifically, a further special type of signal is known to occur. When multiple corneal reflections are available or feature localization in the eye image is otherwise unstable, the tracker may alternate between tracked locations rapidly, causing spiky gaze position signals such as seen in Fig. 3. We will call such signals *high-frequency oscillation* (HFO) in this paper.

### Signal type

As seen in Figs. 2 and 3, gaze position signals can range from smooth to very spiky. We refer to this aspect of the signal as its type. These smooth and spiky gaze position signal types in fact lie along a continuum that can be described by a single parameter, the spectral color of the signal. Spectral color is assessed through Fourier analyses of the recorded signal.

All signals that occur in practice can be decomposed into the sum of a series of periodic (sinusoidal) signals by means of a Fourier transformation (Bergland, 1969). Such analyses can reveal characteristics of the system under study, such as its input-output dynamics (Thomas, 1969), and whether the system’s outputs exhibit serial dependence. There is a long history of applying such analyses to eye-tracking data (e.g., Stark et al., 1958, 1961, 1962; Campbell et al., 1959; Bahill et al., 1981, 1982). Fourier analyses of fixational eye movements have previously been done by studies such as Findlay (1971), Eizenman et al., (1985), Coey et al., (2012) and Bowers et al., (2019). General advice on how to perform such analyses has been provided by Pugh et al., (1987) and Eadie et al., (1995), therefore here we will focus on one specific application of Fourier analysis of eye-movement data: analyses of spectral color using periodograms.

Periodogram analysis yields the power spectral density (PSD) of a signal, revealing the amount of power in the signal in each frequency bin. Assuming the sampling frequency at which a signal is acquired is sufficient (cf. Pugh et al., 1987; Nyquist 1928; Shannon 1949), PSD analysis provides information about the dynamics of the system under study. It is commonly found that dynamical systems follow power-law scaling behavior of the signal’s power with frequency, as described by the formula

8 $$ S(f)\propto \frac{1}{f^{\alpha}}  $$

where *S*(*f*) is the PSD as a function of signal frequency *f*, and *α* a scaling exponent.

*α* is used to assess the spectral color of a signal, and carries information about temporal dependencies in the signal.^2^*α* = 0 signifies a PSD that is flat and has equal power at all frequencies in the signal. Such signals are called white, and do not contain temporal dependencies, meaning that each sample is uncorrelated to all the samples before and after it. Examples of gaze position signals with values of *α* just above zero are given in the middle row of Fig. 2.

An *α* above 0 indicates inverse power-law scaling (also known as 1/*f* signals), which means that the signal shows persistent behavior, as evidenced by a positive correlation between samples (e.g., gaze positions) nearby in time. Such a signal has more power at its lower frequency components and thus fluctuates more smoothly (see top and bottom rows of Fig. 2) than a white signal because nearby samples are, intuitively speaking, attracted to each other. Pink noise is an example of such a signal, referring to *α* = 1. Note however that the term pink noise is sometimes used more loosely as referring to 0 < *α* ≤ 2.

An *α* below 0 indicates anti-persistence in the signal as indicated by negative correlations between samples nearby in time. Such a signal has more power at its higher frequency components and thus fluctuates more violently than a white signal as nearby samples, intuitively speaking, repel each other. An example of such a signal is HFO (see Fig. 3).

## New measures of signal magnitude and signal type in eye-tracking data

Next, we introduce two new measures assessing the magnitude of variability in a signal and the signal’s type. Through simulations later in the paper, we will show that the new signal magnitude measure is relatively stable over different signal types, compared to RMS-S2S and STD. We will furthermore show that the signal type measure we introduce provides a value that typifies a gaze position signal on a continuum ranging from smooth and colored to HFO spikes.

**Fig. 4:**
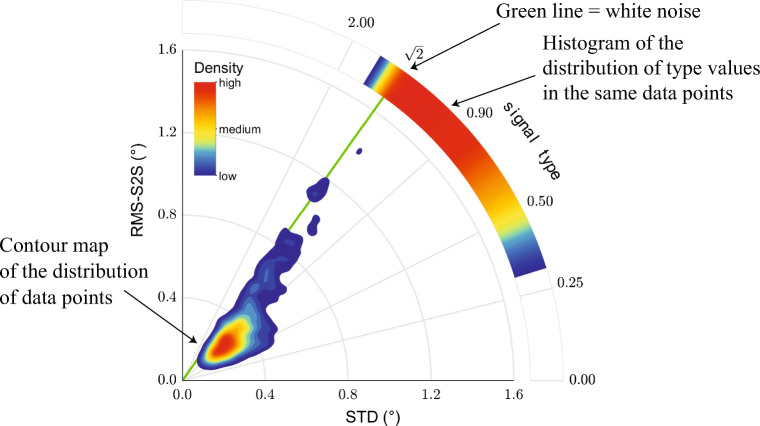
The RMS-S2S – STD space. Signal type (Eq. 10) is represented along the polar axis as the tangent of the direction from the origin to a data point. Signal magnitude (Eq. 9) is the distance from the origin to a data point. This plot is generated by calculating the RMS-S2S and the STD value for each gaze position segment, and then summarizing the data in the space in the form of a contour plot and a histogram. The contour map shows how data are distributed in the RMS-S2S – STD space, while the circular histogram on the polar axis visualizes the corresponding distribution along the signal type dimension

Both measures are constructed from a new representation of variability in eye-movement data, the RMS-S2S – STD space (Fig. 4). In this space, the signal magnitude of a gaze position segment is given by the distance from the origin,

9 $$ \text{signal magnitude} = \sqrt[]{{\text{RMS-S2S}}^{2}+\text{STD}^{2}}  $$

This measure can be interpreted as an index of noise level, weighing both the sample-to-sample step size of the signal and its spatial extent equally. Given that both RMS-S2S and STD are angular extents, signal magnitude is also an angular extent.

The signal type is indicated by the tangent of the direction of a data point in this space,

10 $$ \text{signal type} = \frac{\text{RMS-S2S}}{\text{STD}}  $$

In this paper, we will refer to this measure as *signal type*. The signal type measure can intuitively be likened to the ratio of the displacement between successive gaze positions to the spatial extent of that same segment. The signal type value would be large when the distance between successive gaze position samples spans a large part of the extent covered by a segment of gaze data, such as is the case in HFO signals. On the other hand, a small signal type value would be indicative of a smoothly changing segment of gaze position data. Signal type is a dimensionless quantity, as it is the ratio of two angular extent measures with the same units. We have determined through simulations that signal type values range from 0 to 2, and that white signals always have a signal type value of $\sqrt {2}$.

**Fig. 5:**
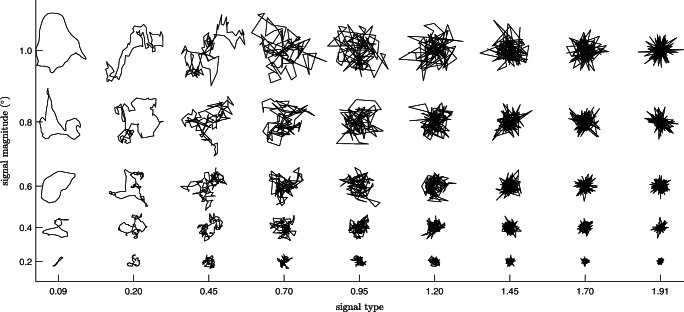
Examples data for combinations of signal type and magnitude. Synthetic data showing what signals (100 data points) of various signal type values look like for various signal magnitudes

Figure 5 shows synthetically generated examples of signals with various type and magnitude values (the synthesis method is presented in the “Noise synthesis” section below). From these figures, it can be intuited that signal type and signal magnitude are measures of independent aspects of a signal. A data segment with a given signal type value can be magnified or shrunk but will retain its signal type value. From Fig. 5, it can clearly be seen that for each signal type value, the type of the signal (from smoothly varying to spiky) is the same for all signal magnitudes. It can also be seen that the spatial extent of the signal remains reasonably constant for a given signal magnitude across all signal type values.

Closer inspection of Figs. 2 and 5 reveals that the signal type value indeed appears to reflect a property of the signal. Low signal type values correspond to smooth gaze position sequences (see the top and bottom rows of Fig. 2 and the left columns of Fig. 5). The right columns of Fig. 5 show that for signal type values close to 2 the signals become spikey, with large sample-to-sample movements back and forth between points at the perimeter of the spatial extent of the signal. Examples of such signals from real data are also shown in Fig. 3. In between these extremes, we find intermediate signal types for the middle signal type values, suggesting that the signal type value is a continuous measure of the signal type in eye-tracker data, from smooth, colored signals to HFO. Indeed, when the signal type value is calculated for a white signal, it results in the value $\sqrt {2}$ (e.g., the middle row of Fig. 2 shows segments of eye tracking data with signal type values just below $\sqrt {2}$, and the third column from the right in Fig. 5 shows segments with values just above $\sqrt {2}$).

## Method

In this paper, the same data set as analyzed in Niehorster et al., (2020b) is used. The detailed methods for data collection and analysis were presented in Niehorster et al., (2020b), here we provide a brief summary.

### Data acquisition

Human data were acquired from four participants completing a fixation task consisting of a sequence of 213 fixation targets, each presented for 1500 ms. Data were recorded separately with five eye trackers, the SR Research Eyelink 1000Plus in desktop mount and head stabilized mode at 1000 Hz, the SMI RED250 at 250 Hz, the SMI RED-m at 120 Hz, the Tobii TX300 at 300 Hz and the Tobii X2-60 at 60 Hz. Using these five eye trackers, data were furthermore recorded from a set of artificial eyes.

### Analysis

For each fixation, for the data from each eye independently, a 200 ms window of gaze position data was selected for which the precision measures were calculated. This window was selected without relying on the above precision measures and such that it was unlikely that it contained microsaccades.

For these windows, a total of six measures, RMS-S2S, STD, $\sqrt {\text {BCEA}}$, the signal type value (Eq. 10), signal magnitude (Eq. 9) and the scaling exponent (*α*) indicating the slope of the PSD, were calculated. Calculation of all these measures except *α* was performed straightforwardly according to the equations presented above. The slope of the PSD *α* was calculated through a line fit to the log-transformed result of a periodogram analysis.

As described in Niehorster et al., (2020b), unfiltered data was acquired for the EyeLink by performing separate recordings for two participants with its heuristic filter switched off, and for the SMI eye trackers by analyzing the gaze vector data provided by these eye trackers in SMI’s headbox coordinate system. For Tobii systems, only unfiltered data was available.

To visualize the distribution of the data in the RMS-S2S–STD space as well as along the signal type continuum (as shown in Fig. 4), the computed RMS-S2S and STD values were collected per eye tracker for all windows of all eyes and submitted to a 2D kernel density estimation procedure (Botev et al., 2010). The output was then discretized into a 16-level contour plot of which the lowest level was removed for clarity.

## Noise synthesis

### Method

For our investigations in this paper, it is necessary to be able to generate artificial noise of different signal magnitudes and signal types. Such a noise synthesizer is also important for testing the robustness of event classification algorithms to noise. Here we present such a synthesizer.

Given the relationship between the signal type value and the scaling exponent *α* of the PSD that will be established below in the “Relationship between signal type and scaling exponent α” section of this article, data with a specific signal type value can be generated by reading off the corresponding *α* value from a graph such as presented in Fig. 9a. Data with this *α* value can then be created by spectrally shaping white noise using standard methods, thereby also achieving the desired signal type value. Using this insight, realistic noise for eye-tracking data with desired signal type and signal magnitude values can be generated with the procedure outlined below. MATLAB code implementing this procedure is available here: https://github.com/dcnieho/FixationalNoise_generator.

1. Generate two *N*-sample white noise signals with a random number generator from a given distribution such as uniform (e.g., using the MATLAB function *rand*), Gaussian (the MATLAB function *randn*), or from a given empirically observed distribution through inverse transform sampling (Devroye, 1986) of that distribution’s CDF. All three methods are demonstrated in the code we provide. One signal will form the horizontal gaze position signal, the other the vertical signal.
2. Fourier transform the generated white noise signals.
3. Spectrally shape the resulting sequences of Fourier coefficients by multiplying them with the amplitude spectrum of 1/*f*^*α*^ noise, where the desired scaling exponent *α* is looked up from a pregenerated table indicating the mapping from *α* to the signal type value for a signal of *N* samples.
4. Inverse Fourier transform the shaped sequences to yield a noise signal with the desired 1/*f*^*α*^ characteristics.
5. Center the resulting signal by subtracting the sequence’s centroid from all samples.
6. If anisotropic noise is desired, scale the horizontal signal by desired ratio of the noise’s horizontal and vertical ranges. If the anisotropic noise should have its major axis oriented in another direction, rotate it.
7. Compute the signal magnitude values of the resulting 2D noise signal, and scale each sample by the ratio of the desired signal magnitude value to the signal’s current signal magnitude value. Scaling with equivalent logic can be performed to achieve a specific RMS-S2S or STD value.

It should be noted that because Fourier transforms are used in this method, the resulting synthetic noise sequences are cyclical in the sense that the last sample of each sequence smoothly connects to the first.

### Example generated signals

Example noise epochs generated with this procedure are displayed in Fig. 5. Generating noise using this procedure enables independent control over four orthogonal aspects of the output signal: its signal type value, its magnitude (signal magnitude, RMS-S2S or STD), its distribution (Gaussian, uniform, or otherwise) and isotropy (its aspect ratio and orientation in the X-Y space). In this paper (e.g., Fig. 5), we have used this method to generate isotropic signals following a Gaussian distribution. In this section, we show further examples of generated signals where the four signal aspects were varied.

**Fig. 6:**
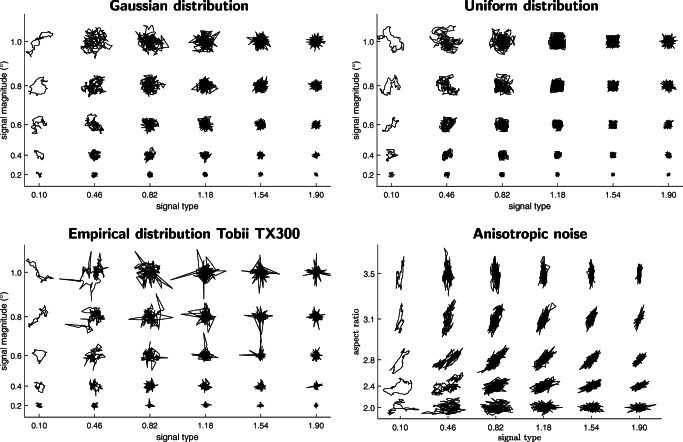
Synthetic data with various properties. Synthetic data (200 samples for each realization) showing what noise following a Gaussian distribution, uniform distribution, or the empirically observed distribution of the Tobii TX300 look like for various combinations of signal type and signal magnitude values. The bottom-right panel shows Gaussian-distributed anisotropic noise generated at several aspect ratios and orientations. For illustration purposes, data in all panels were scaled arbitrarily. Scaling was done with a single common factor across the three isotropic noise panels, so that data in the three panels remain directly comparable

Figure 6 shows examples of generated isotropic noise following a Gaussian distribution, a uniform distribution and the empirically observed distribution derived from data recorded with the Tobii TX300 (chosen as an example). The bottom-right panel of Fig. 6 furthermore shows anisotropic noise drawn from a Gaussian distribution and generated with various orientations and aspect ratios. Like in Fig. 5, each panel shows example output for different combinations of signal type values and signal magnitudes.

Together, these panels demonstrate the four aspects of generated noise that can be varied independently using the noise synthesis code presented in this paper, i.e., signal type, signal magnitude (RMS-S2S, STD or the new signal magnitude measure), signal distribution and anisotropy. Note that identical relationships between scaling exponent *α* and the signal type value were observed for the three signal distributions, confirming that signal distribution is a signal property independent of signal type.

## Results

### Stability of measures across window lengths

**Fig. 7:**
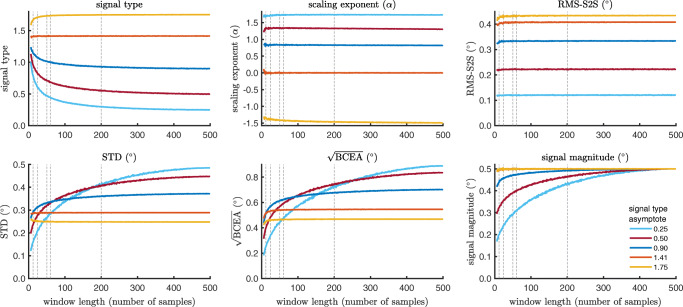
RMS-S2S, STD, $\sqrt {\text {BCEA}}$, signal magnitude, signal type, and scaling exponent *α* values as a function of window length. Generated signals at five asymptotic signal type levels were used to examine how these six precision measures depend on window length. *Dotted lines* indicate the number of samples contained in the 200 ms windows used in this study, for each of the five eye trackers. The number of samples in the analysis window differed across eye trackers due to the different sampling frequencies at which they recorded data

To understand the behavior of the various precision measures, we first investigate how they depend on the length of the data segment from which they are calculated. To perform this analysis, we used sequences of synthesized noise that were generated at five signal type values and a fixed 0.5^∘^ signal magnitude value using the method introduced in the “Noise synthesis” section. The six precision measures were calculated for window lengths ranging from 6 to 499 samples by the following procedure. For each window length, a thousand 500-sample long isotropic noise sequences were generated for each signal type value. For each sequence, a between 6- and 499-samples long window was then randomly positioned within the generated sequence and the six precision measures were calculated for this window. This yielded a thousand values for each precision measure for each window length for each of the five signal type values. Each of the resulting sets of thousand values were then averaged and formed a data point in Fig. 7.

This analysis reveals that while RMS-S2S is mostly stable beyond window lengths of about 30 samples, STD grows asymptotically to the value determined by the simulation parameters for signals with type values ${<}{\sqrt {2}}$ (*α*> 0). That the STD values for these signal types depends on window length explains the reduced range of signal type values observed for small window lengths in the top-left panel of Fig. 7. It furthermore means that the calculated signal magnitude depends on window length for signals with signal type values ${<}{\sqrt {2}}$ (bottom-middle panel of Fig. 7). Further interesting observations are that $\sqrt {\text {BCEA}}$ behaves nearly identically to STD (as would be expected for the isotropic signals used in this simulation) and that *α*, like RMS-S2S, is stable for window lengths of more than about 30 samples. Note however that the generated signals closely follow ideal 1/*f* power-law scaling, so it is not certain that the same stability of *α* across window lengths will be observed for actual eye-tracking data whose spectral content does not follow perfect power law scaling (see the investigations reported in Niehorster et al., 2020b).

The above analyses show that colored signals with *α*> 0, which visually look smooth, increasingly grow in spatial extent with the number of samples up to a certain asymptote. This implies that STD, as well as all other precision measures incorporating a spatial divergence measure, provide different views of the data depending on the window length that is used. What then is the appropriate window length to use for signal evaluations? We do not believe that there is one “correct” window length, but simply that it is required to have an appreciation for the effect that the chosen window length may have on the derived results. For the current paper, our choice of window length (200 ms) is appropriate for our analyses in which we only aim to discover systematic relationships between the measures within each eye tracker.

It is furthermore worth noting that the interaction between window length and signal type when calculating STD (and $\sqrt {\text {BCEA}}$) can lead to an ordering of signal magnitudes that differs depending on window length. This is clearly seen when contrasting STD calculated from the signal with the strongest color (signal type = 0.25, cyan line) to STD calculated from the white signal ($\text {signal type}=\sqrt {2}$, orange line): for small window lengths the STD of the colored signal is lower than for the white signal, while for large window lengths it is higher. This implies that one has to be careful when using STD to compare signal magnitudes across eye trackers with different signal characteristics, because the results may be specific only to the chosen window length. While signal magnitude is seen to preserve the ordering between the signals across window lengths, at small window lengths it also suffers from underestimation of signal magnitude for (strongly) colored signals.

Lastly, as noted, the *α* measure was found to be stable across window lengths whereas the signal type measure was not, due to its dependence on STD. This complicates the relationship between *α* and signal type, making the exact signal type value hard to interpret. Signal type is however a useful measure to quickly ascertain whether a noise signal is white (*α*= 0), *α*> 0 or *α*< 0 using only the easily calculated (or manufacturer-provided) RMS-S2S and STD values of a signal.

### Relationship between measures: Correlational analyses

The understanding that the different precision measures describe different properties of the gaze position signals and the inconsistent rankings between eye trackers shown in Table 1 highlight the need to develop a better understanding of how the six precision measures used in this paper relate to each other. We therefore quantified the extent to which the different measures tap into the same signal properties by means of a regression analysis. Specifically, we assessed the amount of shared variance between each combination of two measures, as measured by a regression model’s coefficient of determination (*R*^2^). Due to the nested nature of the dataset, regression analyses were performed with a multilevel model with the eye from which a data segment was recorded as a random factor. The model contained an intercept and a linear (first order) term for all analyses. *R*^2^ for these multilevel models was assessed by the multilevel variance partitioning method (LaHuis et al., 2014). Where available, human data with the eye tracker’s default filters applied was used for this analysis. For the Tobii TX300 and X2-60 we used the default unfiltered data. The top 1% of precision measure values were removed per eye tracker for each measure before submitting the data to the regression analysis since these last few points exerted large leverage on the fit. For fits involving the RMS-S2S measure calculated from data from the SMI RED250, the top 5% were removed due to a larger number of extreme data points that strongly affected the *R*^2^ measure. Analyses using higher order polynomial terms in the regression model did not reveal any quadratic or higher order relationships between the measures and are thus not reported here.

**Fig. 8:**
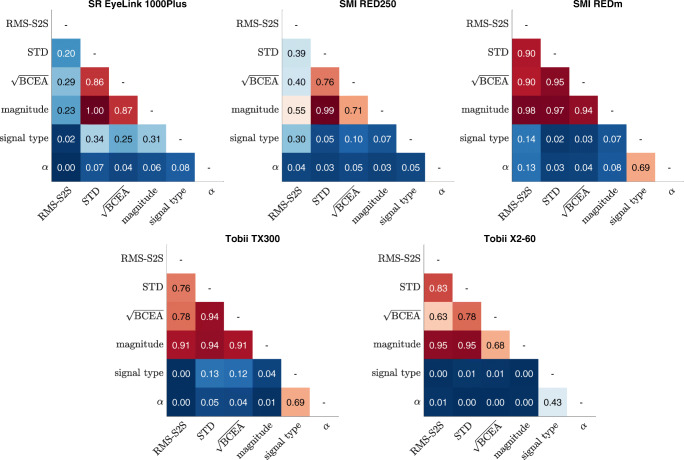
*R*^2^ for linear correlation between measures. Variance explained by a linear relation between the measures RMS-S2S, STD, $\sqrt {\text {BCEA}}$, signal magnitude, signal type, and scaling exponent *α*. Each cell shows the multilevel variance partitioning *R*^2^ value derived using a multilevel regression model fit to the data of all eight human eyes and all 213 fixations

Figure 8 shows the *R*^2^ values between all measures for each of the eye trackers. For all eye trackers, the three measures that reflect the spatial extent of the signal, STD, $\sqrt {\text {BCEA}}$, and signal magnitude, correlate very highly with each other. This indicates that the new signal magnitude measure does not describe a new aspect of the signal beyond that already described by STD and $\sqrt {\text {BCEA}}$. For the SMI RED-m and both Tobiis, RMS-S2S also correlates strongly with STD, $\sqrt {\text {BCEA}}$, and signal magnitude. For the EyeLink, correlation between these measures was only small, while for the SMI RED250 it was moderate. Both signal type and the scaling exponent *α* generally show almost no relationship to any of the other measures except that for three of the eye trackers (SMI RED-m and the two Tobiis), moderate to large correlations are found between the signal type and the scaling exponent *α* measures themselves.

### Relationship between signal type and scaling exponent *α*

Findlay (1971), Coey et al., (2012) and Wang et al., (2016) have previously used PSD analyses to assess the color of gaze position signals. Above we have suggested that the signal type measure also likely reflects the color of the signal, given that it distinguishes between smooth and random signals. Specifically, we expect there to be a systematic relationship between these two measures as both reflect the temporal dependency in the signal. Here we therefore investigate what the relationship between these two measures is. Elucidating this relationship would not only provide an easy shortcut to analyzing the signal color of eye trackers, but would also give end users a simple method to determine the signal nature of an eye tracker from the specification sheet provided by the manufacturer.

We first examined the relationship between signal type and scaling exponent *α* through simulations. Specifically, we generated perfect realizations of 1/*f* (colored) signal sequences with a desired scaling exponent *α* using the synthesizer presented in the “Noise synthesis” section and then computed the signal type value of this sequence. The average of the signal type and *α* measures over 800 such signal sequences was used when examining the relationship for 12- and 24-sample long windows, and 500 sequences for the other window lengths. The lengths of these windows corresponded to 200 ms of data for each of the five eye trackers in this study.

**Fig. 9:**
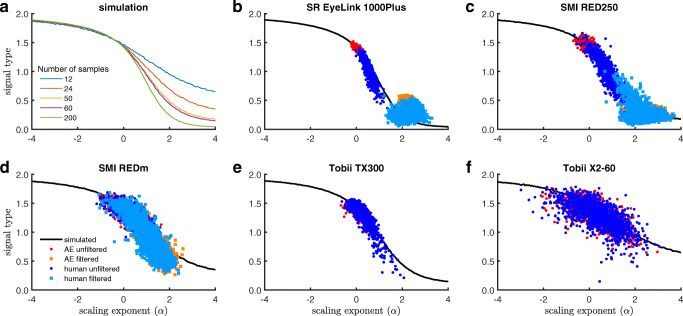
Relationship between scaling exponent *α* of PSD analysis and signal type value. Panel A shows the relationship between the measures derived from simulations employing perfect 1/*f* signals, for different numbers of data points. The other five plots show filtered and unfiltered data from each of five eye trackers collected from artificial eyes (AE) and human eyes (human), along with the relationship between *α* and signal type expected for that eye tracker from the simulation. Note that filtered data were not available for the two Tobii eye trackers

Figure 9a shows the function mapping a given *α* to a signal type value as derived for perfect 1/*f* signals for windows spanning a given number of samples. As can be seen, especially for *α*> 0, the exact relationship between *α* and signal type depends on the number of samples (as shown in the “Stability of measures across window lengths” section above, this is because the STD measure on which the signal type value depends is reduced for small numbers of samples when *α*> 0). The simulation confirms that for white signals (*α*= 0) the signal type is always approximately $\sqrt {2}$.

Next, we examine whether this relationship between *α* and signal type is borne out by the gaze data recorded from humans and artificial eyes. The scatter plots in Fig. 9b–f show for each of the eye trackers the calculated signal type and *α* values of each of the analyzed sample windows in the human and artificial eye data. Where available, both data with the eye tracker’s default filters applied, and data without these filters applied, are shown. It is clear that measures calculated from both filtered and unfiltered data recorded from both humans and artificial eyes align closely with the relationship between signal type and the scaling exponent (*α*) that is expected for each eye tracker from our simulations. This indicates that the mapping between these two precision measures derived through simulation appears to be correct. It should be noted that the relationship derived from the simulation assumed perfect 1/*f* signals, but that the data recorded in practice often deviated from perfect power law scaling (see the investigations of the same data reported in Niehorster et al., 2020b). This may explain part of the variation in the observed data.

Note furthermore that from Fig. 9 it is readily understood why in Fig. 8 a correlation is reported between signal type and *α* for the SMI RED-m, Tobii TX300 and Tobii X2-60, but not for the SR EyeLink 1000Plus and SMI RED250. For the latter two eye trackers, all the filtered human data that were used for the analyses reported in Fig. 8 fall in the tail-end of the signal type–*α* space where the function describing their expected relationship asymptotes and flattens out. As such, no linear correlation would be expected to be found, but the data for the EyeLink and the RED250, like the data for the other three eye trackers, are still close to the values expected from our simulations.

### Example use of the RMS-S2S–STD space plots

**Fig. 10:**
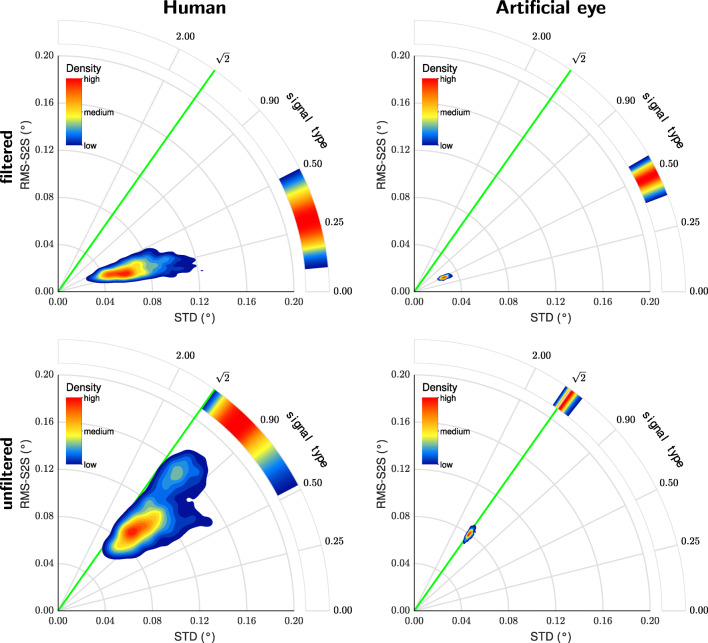
RMS-S2S–STD contour plots for SR EyeLink 1000Plus. RMS-S2S–STD contours plots for filtered (*top row*) and unfiltered (*bottom row*) data recorded from human eyes (*left column*) and artificial eyes (*right column*)

In this section, we will briefly illustrate the use of RMS-S2S–STD space plots to assess signal characteristics of eye tracking data. Plots of this space allow to assess signal type and signal magnitude at the same time. Figure 10 shows example RMS-S2S–STD contour plots derived from the filtered and unfiltered gaze position data recorded from both human and artificial eyes with the SR Research EyeLink 1000Plus. For these plots, only the filtered human data were used from the two subjects for who unfiltered data were also available.

Examining the top row of Fig. 10 shows that the human data were noisier than the artificial eye data (larger distance from the plot origin), but not qualitatively different—the signals recorded from both human eyes and artificial eyes were colored as indicated by signal type values well below $\sqrt {2}$. Detailed inspection however reveals that the data recorded from human eyes were colored more strongly (lower signal type value) than data recorded from the artificial eyes.

Examining the bottom row of Fig. 10 clearly shows that unfiltered EyeLink data recorded with an artificial eye exhibited white signal dynamics (data overlap the green line indicating a signal type value of $\sqrt {2}$), in contrast to the colored signal observed when recording from artificial eyes with the filter switched on. Switching off the EyeLink’s heuristic filter however also led to an increase of the signal magnitude (larger distance from the plot origin). Furthermore, the unfiltered human data were also much whiter (higher signal type value) than when recording from human eyes with the filter switched on, and also had a larger signal magnitude.

This pattern of results has a bearing on the question whether colored signal dynamics in gaze position data are due to fixational eye movements or due to filters in the eye tracker. This question is explored using the current data set in Niehorster et al., (2020b). For our current purpose however, we have shown that RMS-S2S–STD plots provide a quick and simple diagnostic of both the signal type and the signal magnitude of a piece of data. The signal type and signal magnitude values themselves, which are straightforwardly calculated from the RMS-S2S and STD values of a data segment or from an eye tracker’s specification sheet as ${\frac {\text {RMS-S2S}}{\text {STD}}}$ and ${\sqrt []{{\text {RMS-S2S}}^{2}+\text {STD}^{2}}}$ respectively, also provide this opportunity.

### Signal distribution

**Fig. 11:**
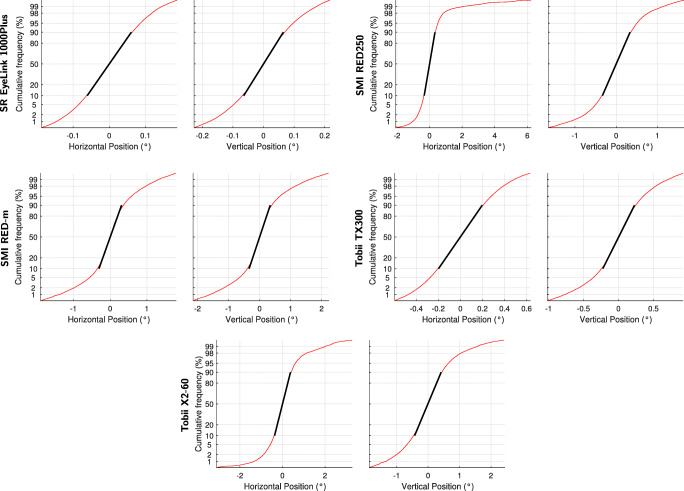
Empirical cumulative distribution function of gaze position data. Empirical CDFs for horizontal and vertical gaze position data, plotted for eye-tracker data against a *z*-scaled (probit) ordinate. The *black lines* present the best fit straight lines covering the central 80% of each empirical CDF

We assessed whether the signals recorded from human eyes during fixation follow a Gaussian distribution through observational means. Specifically, we first centered each of the 200 ms windows of data so that the centroid of the gaze position data in the window equaled (0,0). We then pooled all the individual gaze positions across all windows and eyes for each eye tracker, derived the empirical cumulative density function (CDF) of these gaze positions and plotted this CDF on a *z*-scaled ordinate axis. On such an axis, data following a Gaussian distribution will lie along a straight line whose slope is directly related to the standard deviation of the generating Gaussian distribution. The results for the five eye trackers are plotted in Fig. 11.

As can be seen in Fig. 11, the central part of each of these plots describes a straight line, indicating the gaze position data are mostly well described as coming from a Gaussian distribution. The flattening off at the tails of the CDFs however indicates that the data come from a distribution with heavier tails than a standard Gaussian distribution. To assess what portion of the data is well described by a Gaussian distribution, we fitted straight lines to the 80% most central data points of the CDF in each plot (see the black lines in Fig. 11). As can be seen, deviations from a standard Gaussian distribution are minimal to nonexistent for all eye trackers when examining the central 80% of the empirically observed signal values. As such, using a Gaussian distribution when synthesizing noise for eye-tracking data may be sufficient for most use cases. Where it is desirable to use a full empirically observed signal distribution, this can be easily achieved through the inverse transform sampling technique (Devroye, 1986) using empirical CDFs such as those plotted in Fig. 11. Both methods were described above in the “Noise synthesis” section.

## Discussion

In this paper, we have reported analyses of the gaze position signals recorded with video-based eye trackers during human fixation episodes and in data from artificial eyes. Using these data and several existing precision measures as well as two new measures that we introduced in this paper, we performed investigations with three aims. First, we used simulations to investigate how the various precision measures depend on the number of samples over which they are calculated. Second, we used simulations and the recorded data to investigate how the various precision measures relate to each other and how the traditional measures of signal magnitude, viz. RMS-S2S and STD, interact with signal type. Third, using the insights gained from these analyses, we presented a new noise synthesis method that allows generating realistic gaze position signals with independent control of the type, magnitude, distribution, and isotropy of the signals. Below we discuss the findings of these investigations, and their implications for data quality research and the testing of event detection algorithms for noise resilience.

### Assessing eye-tracker precision

For some kinds of eye-movement research, such as research in fixational eye movements, it is important that the eye tracker used for recording the gaze position data has the highest possible precision (lowest possible noise level), because noise is likely an important factor in the practical spatial resolution of the eye tracker (Holmqvist and Blignaut, 2020). Noise may hide some small oculomotor events or distort the movement profile of these events. As such, it is important to ask the question of what precision measure to use when determining which eye tracker features the lowest noise level.

Assessing the signal magnitude of eye trackers with the aim of determining which is most suitable to record data with for a given project is not a trivial manner. As the work presented here and previous work (Blignaut & Beelders, 2012) has shown, none of the considered precision measures, RMS-S2S, STD, $\sqrt {\text {BCEA}}$ and the new signal magnitude measure we propose in this paper (Eq. 9), provide a complete picture. Furthermore, the decision of which precision measure to use for judging an eye tracker’s performance must take into account the context of the research to be performed. For instance, whereas the RMS-S2S measure is most closely related to variability in the eye-movement *velocity* signal and may thus be appropriate to use when velocity-based event classification algorithms are employed to analyze the data, STD instead reflects spread in the position signal and may thus be most useful when assessing the quality of data that is to be analyzed by a dispersion-based algorithm.

A strong point of RMS-S2S revealed by our analyses is that it does not depend on how many gaze position samples are used in its calculation, making it easier to compare gaze data from eye trackers that record at different sampling frequencies. RMS-S2S is however also strongly affected by filters applied to the signal, which color the signal and smooth it out. Comparing RMS-S2S for gaze position signals recorded with eye trackers that apply no filters, with gaze data from eye trackers that apply different filters or different amounts of filtering is therefore like comparing apples and oranges. The incentives to perform filtering are that it may help in signal interpretation during analysis, and also that it is an easy way for the manufacturer to make their system appear on the specification sheet as having a low noise level. However, we think it is important to recognize that filters never introduce new information into a signal, but only remove (hopefully unwanted) parts. That is, gaze position data can always be filtered further, but cannot be unfiltered. As such, whether to filter the recorded data or not should be a choice that is left up to the user. Among the eye trackers sampled in this study, only the SMIs apply filters that cannot be switched off by the user.

STD, and the very closely related measure $\sqrt {\text {BCEA}}$, are also affected by filters, but less strongly so than RMS-S2S, as indicated by the signal type value decreasing when filters are applied to the eye-tracker signal (see, e.g., Fig. 10). As Fig. 7 shows, filters mostly affect STD for small numbers of gaze position samples. Our results show that the new signal magnitude measure may provide a reasonable composite of RMS-S2S and STD, offering better robustness to changes in signal magnitude due to filtering of the data than RMS-S2S and STD alone. However, Fig. 7 shows that also this measure is affected by filters when it is calculated for a low number of gaze position samples. Nonetheless, this measure potentially offers a better way of comparing eye trackers which produce signals of different types.

How to determine what type of signal is recorded by an eye tracker? Given a piece of data, signal type can be ascertained using *α*, the slope of the gaze position signal’s power spectral density function. When given only the RMS-S2S and STD values of data recorded with the eye tracker, signal type can be assessed with the signal type measure (calculated simply as ${\frac {\text {RMS-S2S}}{\text {STD}}}$). To allow quick assessment of the conditions under which the values on manufacturer specification sheets were derived, as well as identification of the type of signal that is provided by an eye tracker, we therefore urge all manufacturers to report both RMS-S2S and STD values on their specification sheet. Together, these two measures provide a much more complete picture of the eye tracker’s signal quality than either RMS-S2S or STD can provide in isolation.

Can the RMS-S2S measure be used to compare the precision of eye trackers that record at different sampling frequencies? It has previously been argued that the system with a higher sampling frequency has an unfair advantage in this situation (Blignaut & Beelders, 2012; Wang et al., 2016). This argument was developed as follows. Blignaut and Beelders (2012) have reported that when a colored gaze position signal is downsampled, the computed RMS-S2S of this downsampled signal will be higher than when RMS-S2S is calculated for the original signal. The authors showed this effect when downsampling the smooth (colored) signal recorded with an SMI RED250, whereas they found that the computed RMS-S2S remained stable when downsampling the white signal recorded from a Tobii TX300. This has caused them to critique the RMS-S2S measure “because it is applied on sample-to-sample distances - a characteristic that varies with frame rate (if the sampling interval is shorter, the amount of possible movement within the interval is less). (Blignaut & Beelders, 2012, p. 290) Later, Wang et al., (2016) further developed this critique:

With the same movement over time, high-sample-rate system [*sic*] would have better-precision data than low-sample-rate systems, as a result of the temporal proximity (and, given the nature of the behaviors recorded, therefore also spatial proximity) of successive samples. (Wang et al., (2016), p. 951)

It should be noted however that this logic critically depends on the assumption that variability in the gaze position data recorded by an eye tracker during fixation reflects physical eye rotations. In that case, measurement of the same physical eye rotation at a lower sampling rate would logically yield larger sample-to-sample distances. We however deem it unlikely that the eye trackers investigated by Blignaut and Beelders (2012) and most of the eye trackers investigated by Wang et al., (2016) yield gaze position data with a recoverable trace of fixational eye movements, since their noise level is above that required to reliably recover such small eye movements (Holmqvist and Blignaut, 2020). As such, the variability in a gaze position signal of these VOG eye trackers during a fixation is unlikely to reflect the measured motion of a biological structure that undergoes movement constrained by the laws of physics. Instead, it is likely due to measurement noise produced by the eye tracker. As Niehorster et al., (2020b) discuss, the color in the gaze position signals of these systems (and the apparent fixational eye movements in them) that is seen in such noise signals can be due to the effect of filters in these eye trackers.

Although downsampling a colored signal will indeed increase the RMS-S2S computed from this signal while having little effect on its STD, for the above reasons we think this is not because the variability in the gaze position reflects a hypothetical physical eye movement that is the same across eye trackers (cf. the above quotes). Given that the variability for most systems likely solely reflects (possibly filtered) measurement noise, the sample-to-sample distances in the eye-tracker’s output signal are not *necessarily* larger when recording at lower sampling rates. Instead, assuming a similar source of noise at different sampling rates generated at a stage before signal filters are applied, similar RMS-S2S values would be expected across sampling frequencies. It is therefore not unfair to compare the RMS-S2S precision of data recorded from eye trackers at different sampling frequencies, if this practice is done judiciously using only eye trackers that have high enough signal magnitudes to exclude the possibility that fixational eye movements make up a significant part of the signal. However, downsampling the already filtered signals provided by the eye tracker as done by Blignaut and Beelders (2012) for their RED250 data does not accurately simulate the effect of differing sampling frequencies on precision calculated with RMS-S2S if the signal has color.

### Noise for testing event detection algorithms

In this paper, we have shown that eye tracker signals can be described by at least four orthogonal properties: *type*, *magnitude*, *distribution* and *isotropy*. Above, we have presented MATLAB code for producing noise sequences that provides control over all four properties. One important reason for generating realistic eye-movement noise, is to add it to recorded eye-movement data with the purpose of examining how well analysis methods for eye-tracking data perform at different signal magnitudes. Several such analyses are presented in the literature, including Hessels et al., (2016), Zemblys et al., (2017) and Mack et al., (2017). Whereas Zemblys et al., (2017) and Mack et al., (2017) added Gaussian white noise to their data on the assumption that measurement noise is white, Hessels et al., (2016) instead determined the power spectral density of their gaze position signals during fixation and scaled it to produce additive noise at varying magnitudes. One may ask, which of these is the appropriate method to add noise to gaze position data for the testing of analysis methods? As the analyses in our paper reveal, both white signals and the colored signal measured from a specific system are only points on a continuum of signal types. As such, both of the previously used types of noise can be appropriate to add, but neither are sufficient for performing an analysis that generalizes to the signal characteristics of a large selection of VOG eye trackers.

Therefore, a test of an analysis algorithm that is intended to represent the algorithm’s performance on data of many different eye trackers may do well to test the algorithm on the whole range of possible eye-tracker signal types. Is it expected that different types of signal (as measured by the signal type measure or *α*) affect analysis algorithms differently? This remains as an important question for future studies since it goes to the heart of the replicability of results in the eye movement field across different eye trackers and recording conditions. Tests of the behavior of analysis algorithms, such as for instance the resilience of different event classification algorithms to many different signal types, are critical in understanding both the replicability and the generalizability of results derived using these analysis methods. The tools presented in this article, optionally combined with a recently developed method to synthetically produce realistic-looking eye movement data (Zemblys et al., 2018), now make it possible to generate the large amounts of synthetic eye movements signals with any desired signal characteristic that such a test would likely require.

## Footnotes

1 See https://www.tobiipro.com/siteassets/tobii-pro/product-descriptions/tobii-pro-tx300-product-description.pdf/?v=1.0 and https://www.sr-research.com/wp-content/uploads/2017/11/eyelink-1000-plus-specifications.pdf, both accessed 2020-02-13.

2 This follows from the Wiener–Khintchine relationship which states that the PSD of a signal is given by the Fourier transform of its autocorrelation function. From this, it follows that a flat PSD with *α* = 0 has an impulse autocorrelation function at lag 0, and zero correlation at any other time-lag separation, which means that all samples in the signal are independent from each other. Signals that exhibit a 1/*f* PSD will however also have non-zero autocorrelations at other time-lag separations, and such signals can thus be understood to contain temporal dependencies, i.e., correlations between nearby samples. See Schaworonkow et al., (2015, supplementary material).
